# Aging is associated with increased activities of matrix metalloproteinase-2 and -9 in tenocytes

**DOI:** 10.1186/1471-2474-14-2

**Published:** 2013-01-02

**Authors:** Tung-Yang Yu, Jong-Hwei S Pang, Katie Pei-Hsuan Wu, Max J-L Chen, Chien-Hung Chen, Wen-Chung Tsai

**Affiliations:** 1 Departement of Physical Medicine and Rehabilitation, Chang Gung Memorial Hospital, Linkou, Taiwan; 2 Graduate Institute of Clinical Medical Sciences, Chang Gung University, Kwei-Shan Tao-Yuan, Taiwan; 3 College of Medicine, Change Gung University, Kwei-Shan Tao-Yuan, Taiwan

**Keywords:** Aging, Collagen, Matrix metalloproteinase, Tenocytes, Transforming growth factor-beta 1

## Abstract

**Background:**

Most tendon pathology is associated with degeneration, which is thought to involve cyclic loading and cumulative age-related changes in tissue architecture. However, the association between aging and degeneration of the extracellular matrix (ECM) in tendons has not been investigated extensively.

**Methods:**

We examined tenocytes from Achilles tendons taken from rats of three different ages (2, 12, and 24 months). Tenocyte viability was assessed using the 3-[4,5-dimethylthiazol-2-yl]-2,5-diphenyltetrazolium bromide (MTT) assay. Quantitative real-time polymerase chain reaction (PCR) was used to determine the levels of mRNAs that encode type-I collagen, matrix metalloproteinase (MMP)-2 and −9, tissue inhibitor of metalloproteinase (TIMP)-1 and −2 and transforming growth factor (TGF)-β1. Gelatin zymography was used to evaluate the enzymatic activities of MMP-2 and −9. Furthermore, the concentration of TGF-β1 in conditioned medium was evaluated using enzyme-linked immunosorbent assay (ELISA).

**Results:**

The results of the MTT assay showed that the number of viable tenocytes decreased with age. No differences were observed in the levels of mRNAs that encode type-I collagen and TGF-β1 among the three age groups, and the TGF-β1 concentration did not change with age. However, mRNAs that encode MMP-2 and −9 were significantly more abundant in tenocytes from the aging group, and gelatin zymography revealed that the enzymatic activities of MMP-2 and −9 also increased significantly with age. Furthermore, as compared with young group, mRNAs that encode TIMP-1 and −2 were significantly decreased in tenocytes from the aging group.

**Conclusions:**

Activities of MMP-2 and MMP-9 in tenocytes increase with age. This might provide a mechanistic explanation of how aging contributes to tendinopathy or tendon rupture with age.

## Background

Age is commonly associated with increased prevalence of tendinosis and injury [1-3], and degenerative changes are commonly found in the tendons of people over 35 years of age [4]. The most common pathology observed during surgery for chronic painful Achilles tendon is degeneration or tendinosis [2]. In addition, most pathological changes in spontaneously ruptured tendons are degenerative [4].

Little is known about the roles of mechanisms responsible for aging in the degeneration of tendons, but biophysical investigations have implicated a role for imbalanced homeostatic turnover of the extracellular matrix (ECM) of the tendon [5-7]. Accumulated physical damage on the rotator cuff increased cleavage of matrix components in aging tendons [8]. It appears that both insufficient synthesis and increased degradation of ECM might contribute to the mechanical deterioration of tendons.

The degree of ECM breakdown is controlled by the release of matrix metalloproteinases (MMPs) and their inhibition by tissue inhibitor of metalloproteinases (TIMPs) [9]. Several MMPs have been implicated in chronic tendon pathologies, with increased levels of expression of MMP-1, MMP-2, MMP-9, MMP-19, MMP-23 and MMP-25, and decreased levels of expression of MMP-3, MMP-10, MMP-12, MMP-27 and TIMP-2 in either ruptured or painful tendons [5-8,10]. However, there is currently no direct evidence of an association between age and the activities of MMPs. Gelatinases (MMP-2 and −9) cleave soluble type-IV collagen [11], as well as both native and reconstituted type-I collagen [12]. Cyclic strain may increase the levels of both MMP-2 and MMP-9 in horse superficial digital flexor tendons and human Achilles tendons [5]. Moreover, aging enhances this mechanically induced MMP activity [13]. Therefore, it is crucial to investigate whether aging affects the enzymatic activities of MMP-2 and −9 and their physiologic inhibitors, TIMP-2 and −1 directly, as this could ultimately improve our understanding of the mechanism that accounts for the increasing incidence of tendinopathy in aging populations.

The transforming growth factor (TGF)-β gene family consists of at least five homologous genes that encode proteins with a wide range of effects on the differentiation and activity of many cell types [14]. Three homodimeric isoforms (TGF-β1, -β2, and -β3) exist in mammalian cells [15]. Studies reveal that TGF-β1 selectively stimulates the synthesis of connective tissue matrix components both *in vivo* and *in vitro*, to control the formation and degradation of connective tissues [16,17]. These effects might be augmented by reducing the synthesis of proteinases (MMPs) [18-20], or by increasing the expression of tissue inhibitors of MMP (TIMPs) [20,21]. A study on the effects of aging on the synthesis of rabbit fibroblast matrix showed that the fibroblasts from aging rabbits produced significantly less collagen in response to TGF-β1 than fibroblasts from young rabbits did [22]. However, whether aging alters the secretion of TGF-β in tenocytes has not yet been investigated.

The present study was undertaken to assess the effects of aging on the expression of six mRNAs, the enzymatic activities of MMP-2 and −9, and the secretion of TGF-β1 from tenocytes.

## Methods

All procedures were approved by the Institutional Animal Care and Use Committee of Chung Gung Memorial Hospital, Taiwan.

### Primary culture of rat Achilles tenocytes

Tenocytes were obtained from Sprague–Dawley rats, as previously described [23]. The animals were divided into 3 groups by age: young (2 months), middle-aged (12 months), and near senescence (old, 24 months). Samples from passages 2–4, which contained fibroblasts with normal growth rates and shapes, were used. Similar cell densities were used in each group at the start of the experimental process, and all experiments were performed at least in triplicate.

### 3-[4,5-Dimethylthiazol-2-yl]-2,5-diphenyltetrazolium bromide (MTT) assay

Tenocytes from all age groups were cultured, and cell viability was measured by MTT assay both 24 h and 48 h after plating. After the addition of MTT (50 μg/ml), the mixture was incubated at 37°C for 1 h. Next, the MTT solution was discarded, and 1 ml of dimethyl sulfoxide (DMSO) was added to dissolve the formazan crystals. The optical density of the aliquots was measured at 570 nm OD_570 nm_ using a spectrophotometer (VICTOR™X3 Multilabel Plate Reader; PerkinElmer Inc, Waltham, MA). Fold changes in the OD570 nm values for the middle-aged and senescent tenocytes were calculated relative to the values for young tenocytes.

### Isolation of RNA, reverse transcription, and quantitative real-time polymerase chain reaction (PCR)

Tenocytes were lysed by using a guanidine isothiocyanate buffer. Subsequently, total RNA was extracted with phenol and chloroform/isoamyl alcohol (49:1) to remove proteins and genomic DNA. One microgram of total RNA was reverse-transcribed into complementary DNA (cDNA) by incubating it with 200 units of reverse transcriptase in 20 μl of reaction buffer containing 0.25 μg of random primers and 0.8 mM dNTPs at 42°C for 1 h. Quantitative real-time PCR was performed using an SYBR Green and Mx3000P™ QPCR system (Stratagene, La Jolla, CA). Aliquots (20 ng) of cDNA were used for each quantitative PCR, and each reaction was run in triplicate. The primers used are shown in Table 1. Relative gene expressions between experimental groups were determined using MxPro software (Stratagene, La Jolla, CA), and the mRNA that encodes glyceraldehyde-3-phosphate dehydrogenase (GAPDH) was used as an internal control.

**Table 1:** Primer sequences of target genes for real-time PCR

**Target genes**	**Primer sequence**
*GAPDH*	5’ AGTCTACTGGCGTCTTCA 3’ forward
	5’ TTGTCATATTTCTCGTGGT 3’ reverse
*COL1A1*	5’ TACAGCACGCTTGTGGATG 3’ forward
	5’ TTGGGATGGAGGGAGTTTA 3’ reverse
*MMP-2*	5’ GGAAGCATCAAATCGGACTG 3’ forward
	5’ GGGCGGGAGAAAGTAGCA 3’ reverse
*MMP-9*	5’ CCCACTTACTTTGGAAACG 3’ forward
	5’ GAAGATGAATGGAAATACGC 3’ reverse
*TIMP-1*	5’ GCCTCTGGCATCCTCTTG 3’ forward
	5’ CTGCGGTTCTGGGACTTG 3’ reverse
*TIMP-2*	5’ CCAAAGCAGTGAGCGAGAA 3’ forward
	5’ CCCAG GGCAC AATAA AGTC 3’ reverse
*TGF-beta-1*	5’ AGAGATTCAAGTCAAACTGTGGAG 3’ forward
	5’ CCAAGGTAACGCCAGGAA 3’ reverse

Abbreviation: *GAPDH*, glyceraldehyde-3-phosphate dehydrogenase; COL, collagen; *MMP*, matrix metalloproteinase; *TIMP*, tissue inhibitor of metalloproteinase; *TGF*, transforming growth factor.

### Gelatin zymography

The presence of MMP-2 and MMP-9 in conditioned medium was detected using gelatin zymography, which was performed under non-reducing conditions in a 7.5% SDS-polyacrylamide gel containing 2 mg/ml gelatin (Mini-PROTEAN II system; Bio-Rad Laboratories Ltd, Hempstead, UK). Gels were washed in 2.5% Triton X-100 to remove SDS and allow renaturation of MMPs, before they were transferred to a solution containing 50 mM Tris (pH 7.5), 5 mM CaCl2, and 1 mM ZnCl_2_, followed by incubation at 37°C for 18 h. After staining with Coomassie brilliant blue R250 (Bio-Rad Laboratories, Hercules, CA), pro-MMPs and active MMPs were observed as white lysis bands produced by gelatin degradation. To quantify MMP-2 and MMP-9 activities, densitometric analysis was performed using 1D Digital Analysis Software (Kodak Digital Science; Eastman Kodak Company, Rochester, NY). The values of MMP-2 and MMP-9 were normalized relative to viable cell numbers determined from the MTT assay.

### Enzyme-linked immunosorbent assay (ELISA)

An ELISA was used to measure the concentration of TGF-β1 in conditioned medium (culture supernatant) of tendon cells. The medium was aspirated and transferred to the wells of a 96-well ELISA plate that was pre-coated with mouse anti-TGF-β1 antibody (360 μg/ml, 100 μl/well; R&D Systems, Minneapolis, MN) overnight at room temperature, according to the manufacturer’s procedures. The plate was then read using a microplate reader set to measure absorbance at 450 nm (VICTOR™X3 Multilabel Plate Reader; PerkinElmer Inc, Waltham, MA). Recombinant TGF-β1 was serially diluted from 0 to 2000 pg/ml, and the readings were plotted to generate a standard curve. The amount of TGF-β1 production was normalized relative to viable cell numbers determined from the MTT assay after subtracted the value of culture medium.

### Statistical analysis

All data from the MTT assay and densitometric analysis were expressed as mean ± SEM values. The analysis was performed with SPSS 18.0 software for Windows (SPSS Inc., Chicago, IL). Tenocytes among the three age groups were compared using the nonparametric Kruskal-Wallis test. The Mann–Whitney *U*-test was used for comparisons between any two groups. *P* values less than 0.05 were considered significant.

## Results

### Effect of aging on tenocyte viability

**Figure 1:**
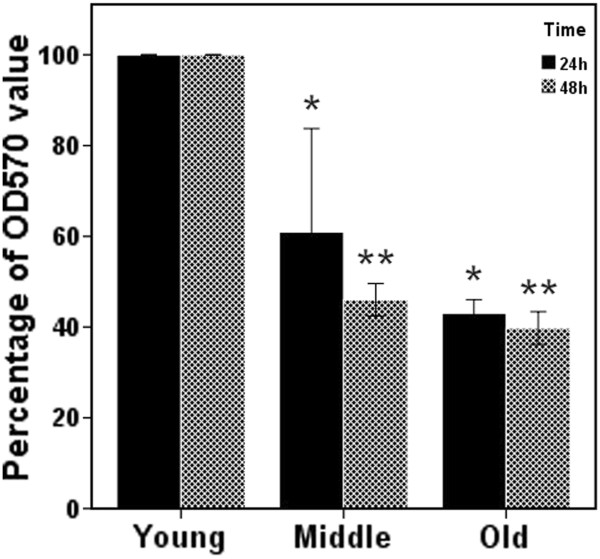
Results of the MTT assay of tenocytes from rats from three age groups 24 h and 48 h after plating revealed that the OD value of old tenocytes was significantly lower than that of young tenocytes (Middle: middle aged; *p < 0.001, ** *p *< 0.001, *n *> 4).

Data from MTT assays revealed that aging lowered the relative OD_570__nm_ values of the aliquots (Figure 1). After 24 h, the respective OD_570__nm_ values of the middle-aged and old rats were 60.9% ± 11.4% and 43.0% ± 1.5% of those of young rats. After 48 h, the respective OD_570__nm_ values of the middle-aged and old rats were 46.0% ± 1.8% and 39.8% ± 1.8% of those of young rats. This result indicated that the viable cell numbers of tenocytes might decrease with age.

### Effect of aging on mRNA expression

**Figure 2:**
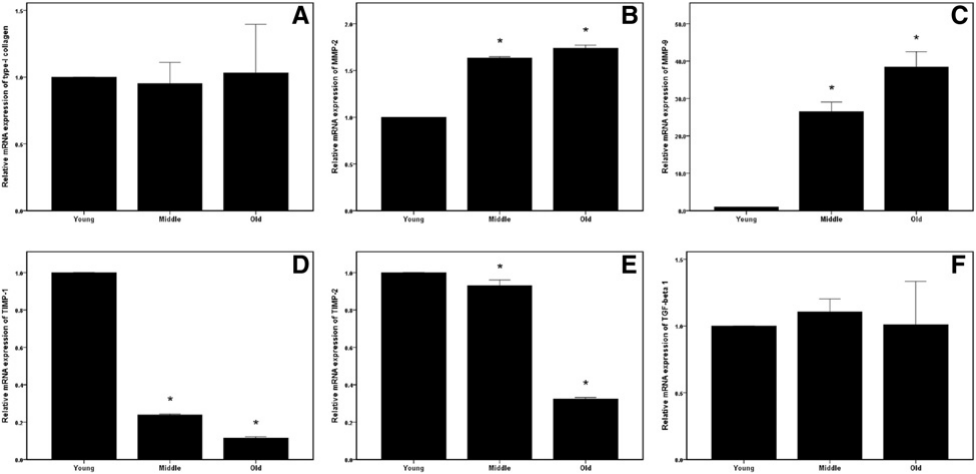
Quantitative real-time PCR revealed: (A) No significant differences among three age groups in the levels of the mRNA that encodes type-I collagen; (B)(C) The level of the mRNA that encodes MMP-2 and MMP-9 increased with age (*p < 0.001, *n *= 3); (D)(E) The level of the mRNA that encodes TIMP-1 and TIMP-2 decreased with age (*p < 0.001, *n *= 3); (F) No significant differences among three age groups in the level of the mRNA that encodes TGF-β1.

Quantitative real-time PCR was used to amplify and simultaneously quantify our target mRNAs. Changes in gene expressions were reported as multiples of increases relative to young rats. Quantitative real-time PCR revealed that levels of mRNAs that encode type-I collagen and TGF-β1 were essentially indistinguishable in tenocytes from young, middle-aged, and old rats (Figure 2A and F). However, *MMP-2* and −*9* mRNA expressions increased significantly with age (*p* < 0.001; Figure 2B and C). Furthermore, as compared with young rats, mRNAs that encode TIMP-1 and −2 were significantly decreased in tenocytes from the old rats (*p* < 0.001; Figure 2D and E).

### Effect of aging on enzymatic activities of MMP-2 and −9

**Figure 3:**
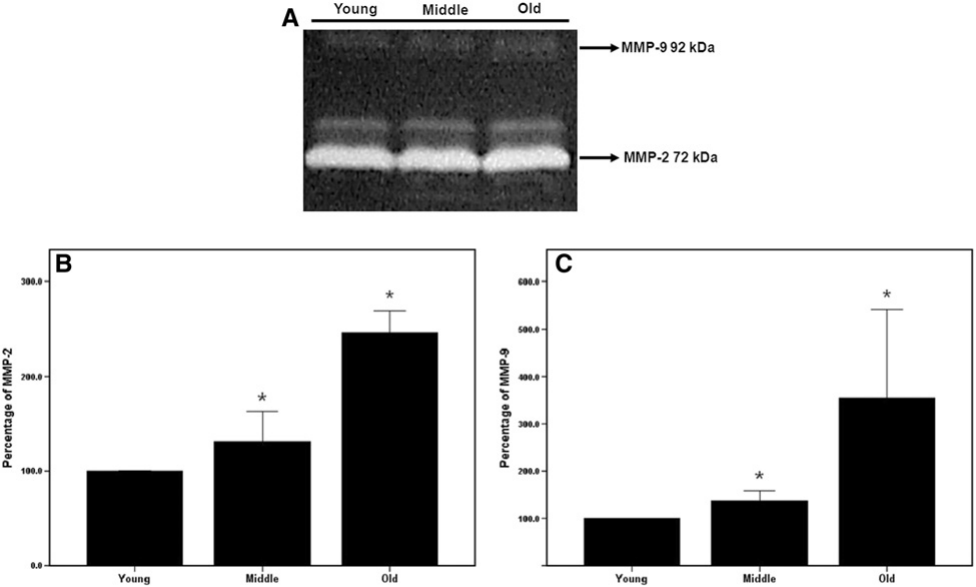
**(A) Zymography of conditioned medium revealed the enzymatic activities of MMP-2 (lower band: 72 kDa) and MMP-9 (upper band: 92 kDa).** (**B**)(**C**) Densitometric analysis of MMP-2 and −9, with levels normalized to the number of viable cells determined using the MTT assay, revealed markedly higher MMP-2 and −9 activity in senescent tenocytes than in the young tenocytes (*p < 0.001, n = 3).

Gelatin zymography analysis of the activities of MMP-2 and MMP-9 revealed that MMP-2 made a greater contribution to the total gelatinase activity in tendon cells than MMP-9 did (Figure 3A). The activities of both MMP-2 and MMP-9 were analyzed for the different age groups by subtracting densitometric readings from the background value and normalizing the data by using the number of viable cells determined using the MTT assay. Senescent tenocytes showed significantly higher gelatinase activities than young tenocytes (*p* < 0.05; Figure 3B and C). This Finding indicates that both MMP-2 and MMP-9 activities increase in an age-dependent manner.

### Effect of aging on TGF-β1 secretion

**Figure 4:**
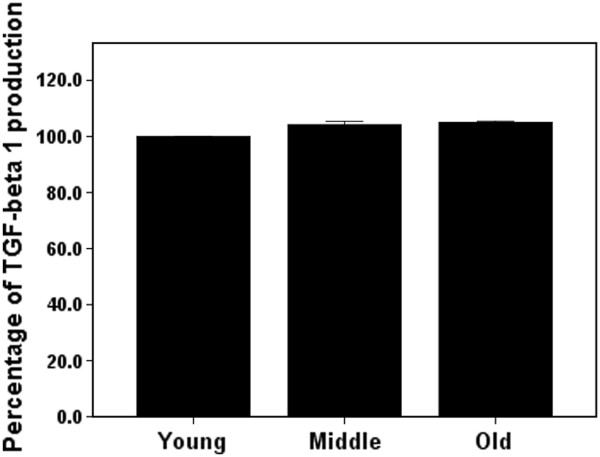
**The percentage of TGF-β1 production in conditioned medium from cultured tendon cells was not affected by the age of the rats from which they were derived *(n *= 3).**

The concentration of TGF-β1 in the conditioned medium were 95.9 pg/ml, 95.8 ± 1.51 pg/ml, 98.9 ± 2.55 pg/ml, and 97.9 ± 1.59 pg/ml for culture medium only and the young, middle-aged, and old tenocytes, respectively. After subtracting the value of culture medium and normalizing the data by using the number of viable cells from MTT assay, the percentage of TGF-β1 production was indistinguishable in the conditioned medium from the tenocytes collected from rats of different ages (Figure 4).

## Discussion

Tenocytes—the basic cellular component of tendons—produce collagens, other proteins, and matrix proteoglycans [24]. Healing of injured tendons proceeds via three overlapping stages: inflammation, regeneration, and remodeling [24,25]. Each stage prepares the healing process for the following stage, so the impairment of one stage may negatively impact the next one. Tenocyte proliferation is one of the principal steps in the regeneration phase of tendon healing. The results of this study indicate that tenocyte viability (which may compose of cell proliferation ability and metabolism) decreases with aging. This might partially account for the poor healing observed in aging tendons. Similar results have been obtained in studies of wound healing, where the proliferative capacity of fibroblast progressively decreases over time [26,27].

Matrix turnover, which involves both the synthesis and degradation of matrix components, is important for the maintenance and repair of tendons. Type-I collagen constitutes around 60% of the dry mass of the tissue and approximately 95% of total collagen [6]. It appears that highly stressed tendons show increased levels of collagen turnover [28]. A study of pathologic human Achilles tendon showed that levels of collagen type-I and -III mRNAs were significantly higher in tendons with chronic pain or spontaneous rupture than in normal tendons [10]. However, the present study demonstrated that aging did not affect the level of the mRNA that encodes type-I collagen. The expression of type-I collagen mRNA is not expected to be a response of aging-related collagen degradation.

The tendon matrix is constantly remodeled throughout life. A relatively high level of matrix remodeling is common in tendons such as the supraspinatus tendon, and this process is linked to degenerative pathology [8]. It appears that MMPs play a key role in regulating matrix remodeling, as they are considered responsible for the degradation of collagens and proteoglycans [7,29]. The results of our present study reveal that the activities of both MMP-2 and MMP-9 are higher in the tendons of aging rats than in the tendons of young rats. To the best of our knowledge, this is the first study to measure gelatinase activities in aging tenocytes. However, a similar age-dependent increase in MMP-2 or MMP-9 activity was reported for samples of the skin, heart, articular cartilage, and even plasma [30-33]. It is reasonable to postulate that tendon degeneration may result from the aging-induced over-expression of gelatinase activity. Regarding TIMPs, our data revealed that both *TIMP-1* and *TIMP-2* mRNA expressions were decreased in old tenocytes, suggesting the activities of MMP-2 and −9 in old tenocytes, under less inhibitory effect from TIMP-1 and −2, may further have a more negative impact on the integrity of tendon matrix. These findings provide a molecular mechanism that accounts for the effect of aging on tendon healing. The over-expression of gelatinase activities may impair the turnover of matrix, which could lead to a qualitatively different and mechanically less stable tendon that is more susceptible to damage.

The transforming growth factor-β is active during almost all stages of tendon healing. During inflammation, TGF-β has a variety of effects on the regulation of cellular migration and proliferation, as well as on the stimulation of collagen production [15]. During tendon synthesis, TGF-β1 significantly promoted the accumulation of *COL1A1* mRNA in cultured tendon fibroblasts [34]. For tendon remodeling, TGF-β1 regulates *MMP-2* expression at the transcriptional and post-transcriptional levels by inducing an early increase in *MMP-2* transcription and an increase in the half-life of *MMP-2* mRNA [21]. It is also thought that TGF-β exerts a selective effect on ECM deposition by modulating the action of other growth factors on metalloproteinase and *TIMP* expression [35]. Increased synthesis of TGF-β1 has also been demonstrated for tendon fibroblasts subjected to strain as well as in tendinosis fibroblast cultures [36,37]. However, our study demonstrated that although aging could increase the activities of MMP-2 and −9, aging is not significantly associated with *TGF-β1* expression. These observations suggest that TGF-β1 does not play a major role in either the aging process related to tendinopathy or the age-related regulation of gelatinase expression.

As for other cell culture models, there are several limitations of the model used in the present study. For instance, the behavior of explanted tendon cells is not identical to the behavior of tendon cells in their natural matrix environment *in vivo *[38,39]. Therefore, one should always be cautious about translating culture data directly to the *in vivo* situation. Further animal studies are needed to assess the physiological relevance of our findings. Aging may alter cell activity, but likely also alters the biochemical environment [40]. It may be speculated that using a reduced level of fetal bovine serum (FBS) in culture medium might better simulate the aging condition. Although the design of the present study did not address the effects of different biochemical environment, in previous investigations, it was clearly shown that there was a decreased proliferation rate when lower level of FBS was used. Besides, independent of the levels of FBS in culture medium, there was a better proliferation in cells from young donors than cells from old donors at all times assessed [41]. Meanwhile, immobilization has been demonstrated for an increase of catabolic process of extracellular matrix by increasing the expression of MMPs [42,43]. It is possible that differences in physical activity between the age groups might partly account for the findings in this study. Further study may be performed to compare the MMPs expression between the effects of inactivity and aging.

## Conclusion

This study demonstrated an age-related increase in the level of gelatinase (MMP-2 and MMP-9) activities and decrease in the mRNA expression of TIMP-1 and TIMP-2 in tenocytes, without any effect of age on the levels of mRNA that encodes type-I collagen or TGF-β1 activity. These results imply that aging might exert a negative effect on tendon structure or its healing process by a mechanism that involves increased MMP-2 and MMP-9 activities, and decreased proliferation of tenocytes. Furthermore, the common growth regulator TGF-β does not appear to affect the aging process in tendons.

## Competing interests

The authors declare that they have no competing interests.

## Authors’ contributions

TY: main investigator, drafting and revision of the manuscript. JHS: main investigator, data interpretation and revision of the manuscript. KPS: data interpretation and proofreading. MCL: statistical analysis. CH: data interpretation. WC: chief supervisor, study design and revision of the manuscript. All authors read and approved the final manuscript.

## Pre-publication history

The pre-publication history for this paper can be accessed here:

http://www.biomedcentral.com/1471-2474/14/2/prepub
